# What animals can teach us about evolution, the human genome, and human disease

**DOI:** 10.1080/03009734.2020.1722298

**Published:** 2020-02-14

**Authors:** Kerstin Lindblad-Toh

**Affiliations:** a Department for Medical Biochemistry and Microbiology, Uppsala University, Uppsala, Sweden;; b Broad Institute of MIT and Harvard, Cambridge, MA, USA

**Keywords:** Canine genetics, comparative genomics, genome sequencing, human genetics

## Abstract

During the past 20 years, since I started as a postdoc, the world of genetics and genomics has changed dramatically. My main research goal throughout my career has been to understand human disease genetics, and I have developed comparative genomics and comparative genetics to generate resources and tools for understanding human disease. Through comparative genomics I have worked to sequence enough mammals to understand the functional potential of each base in the human genome as well as chosen vertebrates to study the evolutionary changes that have given many species their key traits. Through comparative genetics, I have developed the dog as a model for human disease, characterising the genome itself and determining a list of germ-line loci and somatic mutations causing complex diseases and cancer in the dog. Pulling all these findings and resources together opens new doors for understanding genome evolution, the genetics of complex traits and cancer in man and his best friend.

## Introduction

### Human disease—early studies

**Figure 1.**
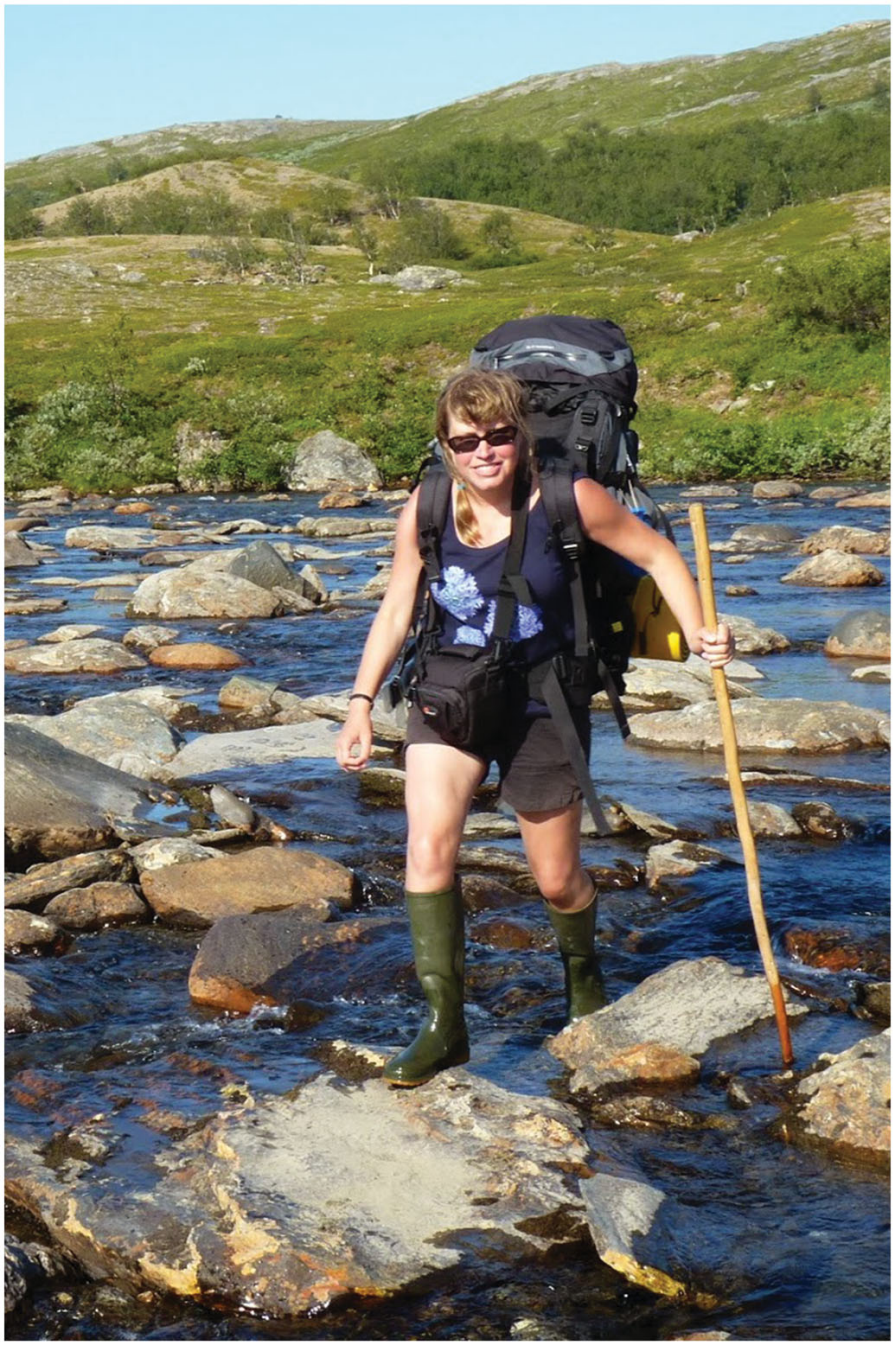
Professor Kerstin Lindblad-Toh, winner of the Medical Faculty of Uppsala University Rudbeck Award 2019, ‘for her excellent research in comparative genomics and for developing the dog as a model for biomedical research’.

Human diseases can largely be divided into infectious diseases and genetic diseases. In many cases diseases arise as a result of both genetic predisposition and environmental factors. In the early years, diseases dependent on a single gene were analysed with laborious methods using large families to see if simple sequence length polymorphism (SSLPs) markers segregated with disease. These single gene diseases included for example cystic fibrosis (*CFTR*) (1) and Huntington’s disease (*IT15*) (2), while Down’s syndrome was found to depend on an extra chromosome (trisomy 21) (3) leading to a more complex phenotype related to many genes. More common complex diseases such as diabetes, schizophrenia, and rheumatoid arthritis were far too complex to understand. On top of this, cancer was postulated to have both inherited mutations and mutations arising in the tumour (including the Knudsen two-hit hypothesis) (4), making the tumour become increasingly malignant (Figure 1).

### Tools have transformed genetics

Over the past 20 years enormous changes related to genome sequencing and gene mapping have occurred, mostly as collaborative efforts striving to develop new technologies, large-scale resources, and computational approaches. As I will discuss below, while DNA was described more than half a century ago, the understanding of how genetic diseases arise is still incomplete, but the field makes continuous progress every day. Many of the tools and analysis methods, and some of the knowledge amassed, are also already being used to understand the biology of disease. Over time, this will allow more detailed diagnosis with better treatment options, and—in the long-term—personalised medicine.

## Comparative genomics

### Sequencing the human genome

The human genome has long been a source of wonder, with only a partial understanding of how it works, despite enormous efforts to generate different types of data. After the discovery of DNA in the 1950s, more active research on the human genome and the genes and regulatory elements hidden therein took a long time to follow. In the beginning, studies often focussed on a single gene for a single disease. Several types of maps relying on different types of markers (SSLPs and radiation hybrid markers) allowed the mapping of monogenic diseases such as cystic fibrosis and Huntington’s disease. It was recognised that the world of disease genetics would greatly improve if the whole human genome could be sequenced. For much of the 1990s, more than 2,800 researchers in a world-wide consortium worked on sequencing the human genome, with different regions or chromosomes divided between labs. This public effort, using Sanger sequencing (5) led to the first human draft genome (6), but also coincided with the publishing of a different human genome (7), which used a novel approach: whole-genome shotgun sequencing, where segments are randomly sequenced and then put together as a giant puzzle. Both genome assemblies covered most of the genome, but struggled with more complex regions such as complex gene families, difficult repeats, and centromeres and telomeres. Thus, the human genome project continued to fill in gaps for an additional 5 years or so. While the human genome was declared finished in 2003 (8), there still remained gaps that were unfilled. In the last couple of years using novel long-read technologies, it has finally become possible to sequence complex regions of the genome such as segmental duplications and repeats, for example in centromeric regions (9), thus enabling new and complete assemblies of chromosomes spanning also repeat regions, from telomere to telomere (10).

Prior to the human genome sequence, common lore had it that the human genome contained ∼100,000 protein-coding genes. When first analysed, the human genome was found to contain ∼50% repetitive sequences, for many years thought of as ‘junk DNA’ (11). In the rest of the genome, scientists struggled to identify the protein-coding genes that must be there. They used available RNA expression data and known protein coding sequence. This allowed them to extrapolate their findings to ∼40,000 protein-coding genes, assuming that there were still genes they could not find. Difficult regions included complex gene families (such as olfactory receptors) or genes with an especially GC-rich sequence.

### Mouse and rat genomes

The second mammalian genome to be sequenced was the mouse (12). The mouse is the best laboratory animal model for human disease, as it is small and easy to manipulate in captivity, and hence it was deemed of high importance to generate a reference sequence for it. For the reference the C57Bl/6J strain was used, despite the fact that multiple strains exist and are used for different disease studies. Intriguingly, the haplotype structure of the mouse genome (as determined by whole-genome sequencing multiple strains) was quite blocky (long stretches of sequences were inherited together when looking at multiple strains), and when multiple strains were analysed and compared to the two founder mice, *Mus musculus domesticus* and *Mus musculus musculus*, it was seen that most laboratory strains were hybrids between those two founder strains (13). This finding agreed with the fact that early mice were used as pet mice based on different phenotypes such as colouring or ‘dancing’ mice in Japan and China (*M. musculus domesticus*) and Europe (*M. musculus musculus*) (14). In addition to studying the genetics in different strains, mice have been used both with knock-out mutations and transgenes. In the current era of CRISPR editing (15), the analysis of mutations in mice has become even easier.

Rats are similar to mice as laboratory animals, but their larger size makes them more expensive to house, while their physiology is more similar to humans. The Brown Norway rat genome was sequenced and published in 2004 (16). Following this, the rat has been widely used to map complex traits by a combination of sequencing and mapping strategies. For example, one study of outbred rats identified 355 quantitative trait loci for 122 phenotypes including anxiety, heart disease, and multiple sclerosis (17).

### Dog genome

The dog, man’s best friend, was the fifth mammal to be sequenced. At 2.4 Gb the dog genome is somewhat smaller than the human genome, based on a lower amount of lineage-specific repeat sequences (334 Mb versus 609 Mb, respectively) (18). Previously, the coding-gene count in mammalian genomes had not been precisely determined, but when using conserved synteny between four mammals—human, mouse, rat, and dog—we could revise the number of mammalian genes to ∼20,000. This number varies slightly between species, primarily based on lineage-specific gene family expansions and contractions, but almost 14,000 genes are 1:1:1 orthologs across human, mouse, and dog (18).

Much research has gone into trying to understand the early history of dogs and wolves. Different studies propose different times and places for the domestication. While the answer is still out there, it would seem logical if wolves domesticated themselves in multiple places and at different times (10,000–40,000 years ago) (19). While studies are ongoing to try to understand the correlation and adaptations making dogs into dogs, one clear genetic event is the duplication of the amylase (*AMY2B)* gene (20), which has only one pair of copies in the wolf, while most dogs have many, roughly five, pairs of copies. This gene is important for the digestion of starch and can be coupled to the changing diet involving considerably more starch in agrarian societies. The fact that dogs living in the Arctic do not have the additional *AMY2B* copies is likely due to their living on a meat-rich diet (21).

### Monodelphis genome and vertebrate evolution

Following the sequencing of a number of useful placental mammals, we moved outside the placental mammals and sequenced the first marsupial — opossum (*Monodelphis domesticata*) (22). The perhaps most intriguing finding when comparing the opossum to placental mammals was that innovations in protein-coding regions are relatively rare, while 20% of eutherian conserved non-coding elements (CNEs) are recent innovations. A substantial proportion of these eutherian-specific CNEs have arisen from sequences inserted by transposable elements (repeats), pointing to transposons as a major creative force in the evolution of mammalian gene regulation.

The chicken red jungle fowl (*Gallus gallus*), being an important food source, was the first bird to be sequenced and published in 2004 (23). The chicken genome is roughly 1 Gb in size, roughly one-third of the human genome, which correlates with a lower number of repeat elements and segmental duplications. In addition to 6 pairs of macrochromosomes, and 1 pair of sex chromosomes (the female is the heterogametic sex), chickens also have 32 pairs of intermediate or microchromosomes. Microchromosomes are small, GC-rich and gene rich. The analysis of the chicken genome in multiple populations has allowed the identification of both morphological traits (24) and traits related to egg and meat production (25)

*Anolis carolinensis* was the first lizard to be sequenced (26). Lizards, like chickens, have substantial numbers of microchromosomes, and they both rely on eggs for reproduction. The evolution of the amniotic egg was one of the great evolutionary innovations in the history of life, freeing vertebrates from an obligatory connection to water, and thus permitting the conquest of terrestrial environments. *A. carolinensis* microchromosomes are highly syntenic with chicken microchromosomes, yet they do not exhibit the high GC and low repeat content that are characteristic of avian microchromosomes. Also, *A. carolinensis* mobile elements are very young and diverse—more so than in any other sequenced amniote genome — which possibly has allowed the novel innovations underlying the rapid radiation of 400 lizard species. These species have radiated, often convergently, into a variety of ecological niches with attendant morphological adaptations, providing one of the best examples of adaptive radiation (27).

Despite the sequencing of a large number of land-living creatures, it was still a mystery how the first creature crawled onto land. Curiously enough, a rarely seen fish, called the coelacanth (*Latimeria chalumnae*), and living in the deep ocean, for example off the East Coast of Africa, was reported to have four lobe-finned limbs similar to many land-living vertebrates. Based on material from a stranded coelacanth, we sequenced the coelacanth genome (28), together with the transcriptome of the lung fish. The lung fish also has four limbs, but as it has an extremely large genome (estimated at 40–100 Gb: http://www.genomesize.com), containing a lot of transposable elements (29), we could not afford to sequence it at that time. Careful analysis of the genes in coelacanth and lung fish showed that the lung fish was more closely related to land-living animals, supporting the primitive notion that both lungs and legs are a great advantage on land.

**Figure 2.**
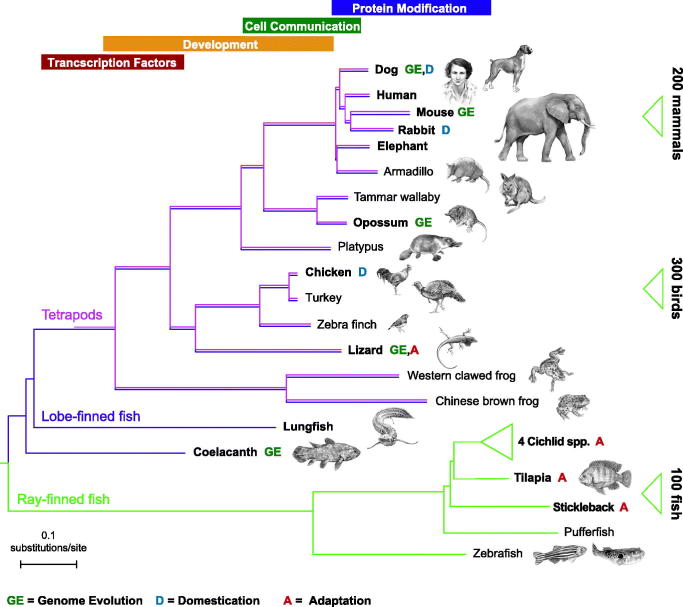
Vertebrate genome sequencing projects shed light on genome evolution, domestication, and adaptation. Many of the first vertebrate whole-genome projects represented model species (e.g. mouse and rat), but over time, additional resources representing natural model species have been added. Highlighted in this tree are some of the studies that have been undertaken, within and across lineages, to study the processes of natural adaptation (marked A; for example, stickleback adaptation to extreme aquatic environments), domestication (marked D; for example, genetic signatures separating domestic dogs and wolves), and genome evolution (marked GE; for example, exaptation changes in a regulatory sequence function between human and monodelphis). As well as indicating the genetic distances between representative vertebrate species, this tree also illustrates the time periods when novel regulatory innovations arose. In particular, regulatory elements near transcription factors (red box) and developmental genes (yellow box) evolved quickly in early vertebrate history, followed by cell communication (green box) and protein modification (blue box) in the more recent past. As whole-genome sequencing becomes substantially cheaper and more accessible, the expansion of reference genomes within each clade is set to increase, with the publication of 200 mammals, 300 birds, and more than 100 fish expected by the close of 2020. Image adapted with permission from Meadows & Lindblad-Toh, Nature Review Genetics (63).

Overall modification of gene-regulatory elements may underlie a significant proportion of phenotypic changes on animal lineages. To investigate the gain of regulatory elements throughout vertebrate evolution we identified a genome-wide set of putative regulatory regions for five vertebrates, including human, and looked for signs of gains. In early vertebrate times regulatory gains occurred frequently near transcription factors and developmental genes, but this trend was then replaced by innovations near extra-cellular signalling genes, and finally, in the last 100 million years (during the mammalian radiation), innovations near post-translational protein modifiers (30). This suggests that the complexity of regulation and function of protein-coding genes have increased continuously (Figure 2).

### Stickleback, cichlids, and herring—good examples of environmental adaptations

Sticklebacks are small fish that were originally marine. They have colonised and adapted to thousands of streams and lakes formed since the last ice age in North America and Europe (31). Typical changes of the freshwater adaptations included body shape, length, depth, fin position, spine length, eye size, and armour plate number. An early study generating a genome sequence also involved the sequencing of 20 individuals from locations spanning across both freshwater and saline environments globally (32). The study identified 90 genomic regions that consistently varied between fresh and salt water. We also noted the re-use of globally shared standing genetic variation, including chromosomal inversions, to allow for repeated evolution of distinct marine and freshwater sticklebacks.

A later study showed similar patterns of adaptation to salinity for the herring, a common food source in Scandinavia, spanning between the brackish Baltic and the salty Northern Atlantic Ocean (33). The genome sequence complemented with whole-genome sequencing of many populations identified >500 regions related to adaptations to brackish water. Further analysis also identified >100 loci that varied between spring and fall breeding populations (34). These studies also suggest that the adaptations can depend on both protein-coding and regulatory adaptations, and that haplotype blocks spanning multiple genes are selected, suggesting that multiple variants in a region might underlie genomic adaptations.

The tilapia, present in the Nile, again a common food source, was used as a backbone for the analysis of the adaptations present in hundreds of cichlid species in the African Lakes of Victoria, Malawi, and Tanganyika. Analysis of four fish from the Eastern lineage showed gene duplications, an abundance of non-coding element divergence, accelerated coding sequence evolution, expression divergence associated with transposable element insertions, and regulation by novel microRNAs compared to the tilapia and other teleost genomes (35). Later studies have also identified strong selection on colour schemes and morphology related to where in the lakes the different species live. Deeper sequencing of five Lake Malawi species followed by genotyping in a diverse collection of ∼160 species from across Africa identified ∼200 genic and non-genic SNPs varying across species. We observed segregating polymorphisms outside of the Malawi lineage for more than 50% of these loci, suggesting that river cichlids have transported polymorphisms between lakes (36).

### Mammals help annotate the human genome

In 2008, sequencing was still relatively expensive, but the whole-genome shotgun approach enabled the use of low-coverage genome sequencing of mammalian genomes to better understand the human genome. In 2011, we published a paper including 2× sequencing (whole-genome shotgun sequencing where random sequences are generated so that each base in the genome is sequenced on average 2-fold) of 18 mammals added to the 11 existing (Sanger sequenced at 7×) mammals (37). This project allowed the identification of evolutionary constraint of 12-bp elements, resulting in the identification of >3 million constraint elements, encompassing 4.2% of the genome. The protein-coding sequence only covers ∼1% of the genome, thus suggesting a flood of novel candidate regulatory elements. The data also allowed us to look at synonymous constraint elements where regulatory elements overlap coding sequence, constraint patterns in promoters, and accelerated regions in humans and primates—hallmarks of positive selection for human adaptations. Recent work has shown that human accelerated elements encompass regulatory elements such as well conserved enhancers for developmental genes (38).

### All is not protein-coding genes

In addition to protein-coding genes (1%) other regulatory entities encompass at least three times as much space. These regions include non-coding RNA transcripts, such as thousands of long intergenic non-coding RNAs (lincRNAs) (39) and microRNAs (miRNAs) (40). LincRNAs are RNA molecules larger than 200 nucleotides and are more or less conserved across species, presumably varying in the strength of function. Still, they have a widespread role in gene regulation and other cellular processes including cell-cycle regulation, apoptosis, and establishment of cell identity (41). MiRNAs are short (20 to 24 nucleotides), non-coding RNA molecules composed of a single-stranded sequence. They predominantly act as negative regulators of gene expression (40), but are functionally involved in virtually all physiologic processes, including differentiation and proliferation, metabolism, hemostasis, apoptosis, and inflammation.

To better catalogue the regulatory landscape, the Encyclopaedia of DNA Elements (ENCODE) project (https://www.encodeproject.org) was formed to map functional non-coding elements. Initially, ChIP-seq was performed to detect the location of individual transcription factor binding sites in many tissues. Over time, this analysis has expanded to look at differential methylation and acetylation of genomic bases and their binding proteins. Recently, Assay for Transposase-Accessible Chromatin using sequencing (ATAC-seq) (42) has been developed to detect open chromatin. Moreover, the 3 C technology has been developed into HiC (43), which can detect topologically associating domains (TADs) allowing scientists to infer what portions of the genome are within specific regulatory regions in specific tissues (44). In addition, the GTEx effort (https://www.gtexportal.org/home/) works to couple genome variation, such as single nucleotide polymorphisms (SNPs), to differences in gene expression.

### More than 240 mammals for single base resolution constraint in mammalian genomes

As Illumina sequencing became affordable, we put together a project with the goal of detecting human single base evolutionary constraint using >240 mammalian genomes (45). Of these, 131 genomes were generated by us using DISCOVAR-*de novo* (46), combined with the 110 mammalian genomes in NCBI in March 2017. As this data set is analysed it will allow the study of: 1) the largest Eutherian nuclear genome phylogeny; 2) the ability to perform genotype–phenotype correlations across many mammalian species; 3) the evolution of genome structure; 4) reference genomes that can be utilised for species conservation; and, finally, 5) a detailed map of evolutionary constraint, which can be used with human genome-wide association (GWAS) catalogues and other species data sets to investigate patterns of constraint in disease-associated regions in any of the 241 genomes. The data set will also make possible the study of accelerated regions under positive selection in any of the sequenced mammalian genomes.

## Comparative genetics

### Complexity of human disease genetics

After the generation of the human genome, lots of effort went into detecting variation in the human genome. SNPs, indels, and larger structural variations were discovered, and SNPs were put into genotyping panels to allow for genome-wide association studies (GWAS) to map disease loci. The initial thoughts were that common diseases were caused by common variants. Although tens of thousands of patients and controls have been genotyped for many complex traits, the loci identified have only accounted for a fraction of the heritability of the disease. As an example, 113,075 controls and 36,989 cases with schizophrenia have identified 108 genome-wide significantly associated loci (47). These loci are estimated to explain 30–50% of the heritability for schizophrenia. There could be multiple reasons for why only a smaller portion of the heritability has been detected, such as the need of larger samples sizes, environmental factors, epigenetics or a bigger proportion of rare variants. For some diseases such as autism, where individuals with the disease reproduce less frequently, the fraction of novel mutations or rare variants of high effect may be larger (48). To detect individual rare variants, much larger sample sizes are needed; however, methods of Burden testing of specific gene regions or pathways will enable the summing of multiple mutations. While currently the gene plus a unified flanking region is involved in the analysis, TAD domains and GTEx data should be helpful in performing Burden tests on regions distinctly important to the gene.

### The dog as a model for human disease

**Figure 3.**
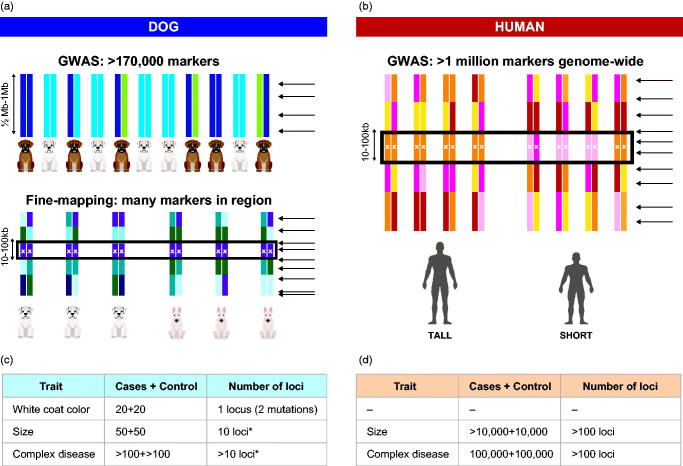
Genome-wide association (GWAS) is easier in dogs than in humans. Monogenic traits in dogs can be mapped with fewer SNP markers and fewer individuals than in humans. GWAS in dogs will utilise the long linkage disequilibrium (LD) within dog breeds, followed by fine-mapping in multiple breeds with the same phenotype (panel a). In humans the LD is short, requiring the use of a lot of SNP markers already in the GWAS step (panel b). The number of SNP markers required for different types of traits in dogs is lower, as is the number of loci contributing to each trait in dogs (panel c), while in humans most traits are more complex and require more samples (panel d).

The strongest reason for sequencing the canine genome was to harness the genetics related to the enormous diversity among breeds (49). Pet dogs are special because they share our environment, and also share the same diseases as humans, including autoimmune disease, neurological disease, cardiovascular disease, and cancer. In fact, roughly 30% of dogs get cancer, which is similar to the frequency observed in man (50). On top of this, there has been very strong selection for morphological traits and behaviour, suggesting that rare variants with strong effect may have become more common in certain breeds, leading to disease. The bottlenecks at breed creation may also have allowed drift to make some alleles much more common in some breeds. The recent creation of breeds (in the last 200 years) also means that haplotype blocks are long within breed and short across breeds. This allows for a mapping strategy where first high-risk breeds are used to find the rough locations of disease mutation (by case control GWAS), and then other breeds are added in to fine-map the region to find the functional mutation (Figure 3) (18). Based on this, monogenic traits can be mapped with ∼20 cases and 20 controls, while complex traits, such as cancers, can be mapped with a few hundred cases and controls. In humans, many thousands of patients and controls are needed. Many diseases such as osteosarcoma (51), canine systemic lupus erythematosus (52), and canine compulsive disorder (CCD) have now been identified and, in several cases, been translated to the corresponding human disease.

### Obsessive-compulsive disorders (OCD) shares a common aetiology between dogs and humans

CCD shows strong clinical similarities with human OCD; both species perform certain normal behaviour in excess and often repetitively. To investigate the genetic causes of CCD, we first performed GWAS in 92 cases and 68 controls of the Doberman Pinscher breed, identifying a single genome-wide significance locus (53). This locus was near the cadherin 2 (*CHD2*) gene, for which the protein is located in the synapse. Secondly, careful reanalysis of the data identified multiple regions of suggestive association as well as regions of fixation in the Doberman Pinscher breed. Thirdly, we performed targeted resequencing of all these regions and identified a number of genes with increased numbers of mutations in cases versus controls (54). Many of these genes were active in the synapse. Finally, we used the genes and pathways found in dogs, combined them with known functionally important OCD genes from humans and mice, and used the combined gene set to perform targeted sequencing of human OCD cases and controls (55). Altogether, we analysed 608 genes in 592 cases and 560 controls and identified four genes as strongly associated (one genome-wide). Two of these genes, *NRXN1* and *HTR2A*, were enriched for protein-coding mutations in cases, while two genes, *CTTNBP2* (synapse maintenance) and *REEP3* (vesicle trafficking), had only regulatory mutations in this study. This might suggest that these two proteins with regulatory mutations have such critical functions that they cannot tolerate coding mutations. Now larger GWAS studies are being performed in humans, and it will be interesting to see if the link to CCD will be further strengthened.

## Taking the next step

### Sequencing technologies change the way we can analyse most species on earth

As the cost of long-read sequencing technologies is finally coming down for generating a high-quality genome (and the generation of population data can be cheaply generated by short-reads), the ability to generate genomes from many species changes dramatically. However, one of the challenges still remaining is access to high-quality DNA samples, which is necessary for generating a reference genome with long-read sequencing, and also for samples from a sufficient number of individuals to allow the generation of population data from different regions of the world. Multiple zoos (i.e. the San Diego Frozen Zoo) have collected samples and cell lines which are potentially useful for generating genome sequences or studying variation, but also potentially for *in vitro* reproduction for endangered species. One such example is the Southern White Rhino which is close to extinction, with only two individuals still alive and neither of them able to carry a pregnancy. Attempts will now be made for a Northern White Rhino to be a surrogate mother (56).

About a year ago the Earth Biogenome Project (https://www.earthbiogenome.org) (57) started with the enormous aim of generating high-quality genomes for each of 10–15 million eukaryotic species on Earth in the next 10 years. To accomplish this, almost every step of the procedure needs to be scaled up: sample collection, sequencing and assembly, annotation, and standardised analysis, as well as species-specific analysis. On top of this comes the generation and analysis of population data and transcriptomics for annotation of genomes. Although this is still a moonshot, we are getting closer. Importantly, to save diversity of life on Earth, sequencing must be combined with more practical conservation efforts such as protection of habitats and inhibition of poaching.

### Mammalian constraint and its use for understanding disease in many mammals

As mentioned earlier, most GWAS loci fall outside protein-coding regions of the genome, thus requiring the use of various ways to annotate single variants for the likelihood that they are a causative mutation for disease. Several techniques are being developed to address this crucial issue. The 200-mammals-data reaches single base constraint for all bases in the human genome, allowing a triage of each position’s likelihood to be functional without any relationship to in which tissues the affected gene is located. As novel methods such as massively parallel reporter assays and genome editing at large scale (58) become possible, it will allow the comparison of overall evolutionary functional constraints to that of functionality in individual tissues.

To add to this, additional annotations of all types of transcripts in many healthy and disease tissues as well as many types of functional annotations (both experimental like ChIP-seq, Regulome and HiC, or bioinformatic Hidden Markov Models [HMMs] (59) and machine learning methodologies) will aid our understanding of how normal and diseased tissues are affected by each gene/mutation. To more clearly understand the tissue-specific effects, single cell sequencing (60) has also become more frequent and can decipher cells in, for example, the immune system and brain, where a large number of different cell types live in close proximity and symbiosis. Cultured organelles and spatial transcriptomics (61) in key tissues allow further dissection of transcriptomics and functional effects.

### Utilising the canine model for clinical trials

The clinical similarity between disease in dogs and humans has been studied for many years. Based on Multiple GWAS data sets, tumour mutations and expression data have shown also a molecular similarity between dogs and humans in many diseases. Based on the shorter life-span in dogs compared to humans, the outcome from clinical trials is likely to be informative more quickly. Mouse models on the other hand, may give rapid results, but are often induced and are rarely spontaneous. Already, trials for canine ALS (https://vhc.missouri.edu/small-animal-hospital/neurology-neurosurgery/current-clinical-trials/) and multiple cancers are underway (https://trials.vet.tufts.edu/clinical-trials/?fwp_species=dog&fwp_veterinary_specialties=oncology). Also, the analysis of cell free DNA (cfDNA) and circulating tumour DNA (ctDNA) for monitoring disease progression in liquid biopsies (62) in the dog should be informative.

## Conclusion

During the past two decades, the understanding of vertebrate evolution as well as of the human genome, and consequently human disease, has expanded at an exceptional pace. We have increased our understanding of evolutionary principles and the content of the human genome. Loci associated with a specific human disease can be in the hundreds, detected with tens of thousands of individuals, yet explaining only a fraction of the disease risk. Thus, we have still only scraped the surface when it comes to understanding human disease.

The next decade is likely to generate exponentially more data to help protect endangered species by having both a reference genome and population data. It will also increase the understanding of the human genome, including non-coding mutations and rare variants. This will require both an understanding of every base in the human genome as well as large sample sizes to fully map human disease. Increasing use of pet dogs for disease gene identification as well as for clinical trials is likely to help propel the biological understanding into canine and human personalised medicine.
